# School principals’ COVID-19 health literacy and its association with mental health: international findings from 11 countries in Europe and Asia

**DOI:** 10.1093/heapro/daag098

**Published:** 2026-07-17

**Authors:** Marlene Meyer, Kevin Dadaczynski, Melanie Messer, Rune Müller Kristensen, Venka Simovska, Areti Lagiou, Evanthia Sakellari, Padmore Adusei Amoah, Sam S S Lau, Angela Y M Leung, Guglielmo Bonaccorsi, Chiara Lorini, Veronica Velasco, Mariusz Duplaga, Karina Leksy, Madalina-Adina Coman, Anita Sandmeier, Cheng-Yu Lin, Tuyen Van Duong, Fatma Özlem Özturk, Pınar Soylar, Emily Marchant, Orkan Okan

**Affiliations:** WHO Collaborating Centre for Health Literacy, TUM Health Literacy Unit, Department of Health and Sport Sciences, TUM School of Medicine and Health, Technical University of Munich, Am Olympiacampus 11, 80809 Munich, Germany; Department Sport and Health Sciences, University of Potsdam, Karl-Liebknecht-Str. 24-25, 14476 Potsdam, Germany; Center for Applied Health Science, Leuphana University Lueneburg, Wilschenbrucher Weg 84a, 21335 Lueneburg, Germany; Institute of Nursing Science, University Hospital Würzburg, Josef-Schneider-Str. 2, 97080 Würzburg, Germany; Department of Nursing Science, University of Würzburg, Josef-Schneider-Str. 2, 97080 Würzburg, Germany; Danish School of Education, Aarhus University, Tuborgvej 164, DK-2400 Copenhagen NV, Denmark; Danish School of Education, Aarhus University, Tuborgvej 164, DK-2400 Copenhagen NV, Denmark; Laboratory of Hygiene and Epidemiology, Department of Public and Community Health, University of West Attica, 196 Alexandras Avenue, 11521 Athens, Greece; Laboratory of Hygiene and Epidemiology, Department of Public and Community Health, University of West Attica, 196 Alexandras Avenue, 11521 Athens, Greece; School of Graduate Studies, Lingnan University, 8 Castle Peak Road, Tuen Mun, Hong Kong, SAR, China; Research Centre for Environment and Human Health, School of Continuing Education, Hong Kong Baptist University, 8 On Muk Street, Shek Mun, Shatin, New Territories, Hong Kong, Hong Kong Special Administrative Region, China; College of International Education, School of Continuing Education, Hong Kong Baptist University, 8 On Muk Street, Shek Mun, Shatin, New Territories, Hong Kong, Hong Kong Special Administrative Region, China; School of Nursing, Hong Kong Polytechnic University, Hung Hom, Kowloon, GH528, Hong Kong, SAR, China; Department of Health Science, University of Florence, Viale G.B. Morgagni, 48 - 50134 Firenze, Florence, Italy; Department of Health Science, University of Florence, Viale G.B. Morgagni, 48 - 50134 Firenze, Florence, Italy; Psychology Department, Università Degli Studi di Milano-Bicocca, Piazza dell'Ateneo Nuovo 1, 20128 Milan, Italy; Department of Health Promotion and e-Health, Institute of Public Health, Faculty of Health Sciences, Jagiellonian University Medical College, Skawinska Str. 8, 31-066 Krakow, Poland; Institute of Pedagogy, Department of Social Science, University of Silesia in Katowice, Bankowa Stret 12, 40-007 Katowice, Poland; Department of Public Health, College of Political, Administrative and Communication Sciences, Babeș-Bolyai University, No. 7 Pandurilor Street, 9th floor, Cluj-Napoca 400095, Romania; Schwyz University of Teacher Education, Zaystrasse 42, 6410 Goldau, Switzerland; Department of Radio, Television and Film, Shih Hsin University, No. 1, Ln. 17, Sec. 1, Muzha Rd., Wenshan Dist., Taipei City 116, Taipei 11642, Taiwan; Department of Public Administration, National ChengChi University, No. 64, Sec. 2, Zhinan Rd., Wenshan Dist., Taipei City 116, Taipei 116-05, Taiwan; School of Nutrition and Health Sciences, Taipei Medical University, No. 250, Wuxing Street, Taipei 11031, Taiwan; Department of Nursing Altindag, Ankara University Faculty of Nursing, Hacettepe Mahallesi, Plevne Caddesi, No: 7, PK: 06230 Altindag, Ankara, Türkiye; Health Sciences Faculty, Department of Nursing, Fırat University, 23119 Elazig, Türkiye; Department of Education and Childhood Studies, Swansea University, Singleton Park, SA2 8PP Swansea, United Kingdom; WHO Collaborating Centre for Health Literacy, TUM Health Literacy Unit, Department of Health and Sport Sciences, TUM School of Medicine and Health, Technical University of Munich, Am Olympiacampus 11, 80809 Munich, Germany

**Keywords:** COVID-19, health literacy, well-being, psychosomatic complaints, school principals, mental health, cross-country analyses

## Abstract

School principals are prone to mental health problems due to work demands. During the COVID-19 pandemic, school principals were additionally tasked with managing challenging responsibilities, i.e. implementing hygiene regulations, communicating frequent policy updates, and transitioning to online teaching. This international study aimed to assess school principals’ COVID-19 health literacy (HL) levels as a resource during the pandemic and to examine associations with well-being and psychosomatic complaints. The COVID-19 Health Literacy School Principal Survey was jointly conducted in 11 countries during 2021 and 2022. *N* = 8644 school or vice principals completed the online survey. Well-being was assessed using the WHO-5 Well-Being Index, psychosomatic complaints with a subscale of the Burnout Assessment Tool and self-reported COVID-19 HL through the HLS-COVID-Q22. HL levels were computed using Rasch analysis. Multiple linear regression analysis was conducted to investigate the association of school principals’ COVID-19 HL with well-being and psychosomatic complaints while controlling for sociodemographic and school-level factors. Overall, 0.1% of school principals had insufficient COVID-19 HL, 3.3% problematic, 65.7% sufficient, and 30.9% excellent. COVID-19 HL significantly predicted well-being and psychosomatic complaints even after controlling for sex, age, position, school type, teaching load, and working hours. Despite the small explained variance, the findings emphasize a need to invest in school principals’ HL as a resource during times of crisis and beyond. Health promotion interventions should be developed to enhance HL and hence health, especially of female and younger school principals, who reported lower well-being and more frequent psychosomatic complaints.

Contribution to Health Promotion

- This study provides the first international, cross-country evidence on the role of school principals’ health literacy (HL) as a resource for personal health-related outcomes.
- School principals with higher COVID-19 HL had higher well-being and less psychosomatic complaints during the pandemic, a time of crisis leadership.
- HL is a modifiable determinant of health. Enhancing school principals’ HL may thus benefit not only their personal mental health but also the health of the whole school, given their pivotal role in enabling health promotion.

## Background

Emerging in 2019 and persisting for several years, the COVID-19 pandemic profoundly affected global health, disrupted economies, and impacted the everyday lives of billions of people (Naseer *et al*. 2023). The negative effects resulting from approaches to address the challenges imposed by the pandemic on the education sector are well documented and include, e.g. planning and implementing hygiene measures, engaging in active health communication, or enabling online teaching (Betthäuser *et al*. 2023, Dadaczynski *et al*. 2021a, Sing Yun 2023, Tri Sakti *et al*. 2022). Detrimental effects on mental health, teaching and learning, quality of life, and physical health were reported for different populations within the school context, including students, teachers, parents, and school administrators (Tri Sakti *et al*. 2022). School principals play a pivotal role not only in planning, governing, leading, and implementing educational processes, but also in shaping school environments that promote health and prevent disease transmission (Dadaczynski *et al*. 2020b; Öztürk *et al*. 2023). Their actions may therefore exert both direct and indirect effects on student, teacher, and their personal health outcomes. Given the significance of their role for school health and public health more broadly, school principals’ health warrants greater attention and should be considered an important target for health promotion and intervention efforts (Dadaczynski *et al*. 2020b). Although recent years have seen increasing interest in this topic, empirical evidence on the health of school principals remains limited (Dadaczynski *et al*. 2022a). Considering that teachers’ anxiety, depression, and stress levels rose due to challenges linked to the impact of the pandemic (Baker *et al*. 2021, Ozamiz-Etxebarria *et al*. 2021), it is plausible to assume that school principals’ mental health was similarly affected, especially given their additional leadership responsibilities during the pandemic (Dadaczynski *et al*. 2021b). A recent scoping review identified a heavy administrative workload, lack of support, and the COVID-19 pandemic as key stressors for school principals (Tahir *et al*. 2026). The COVID-19 pandemic has been classified as a period of ‘crisis leadership’ for school principals, characterized by time-critical decisions, additional tasks and responsibilities, and skill demands beyond routine school operations (McLeod and Dulsky 2021, Smith and Riley 2012).

Studies preceding the pandemic reported associations between school principals’ personal mental and physical health and work-related stress and resources (Dadaczynski *et al*. 2020a, Phillips *et al*. 2008). In an Australian study, school principals exhibited higher levels of burnout and lower well-being relative to the general population (Maxwell and Riley 2017). Consistent with these findings, burnout is commonly observed in school principals (Rogers *et al*. 2025). Even prior to the pandemic, school principals reported high levels of work-related stress, accompanied by substantial turnover in the profession (DeMatthews *et al*. 2021, Marchant *et al*. 2024). Burnout effects and the burdens experienced during the pandemic are likely to have adverse consequences for their health and turnover rates. Maslach and Jackson (1981) originally conceptualized burnout in the working population as comprising emotional exhaustion, depersonalization, and low sense of personal accomplishment. Psychological distress and psychosomatic complaints have meanwhile been identified as secondary symptoms, in that they also manifest in individuals without burnout (Schaufeli *et al*. 2020). The risk of burnout increases with an increasing number of psychosomatic symptoms (Hammarström *et al*. 2023). Psychosomatic complaints are not attributable to physical pathology but rather to psychological problems, typically manifesting as, for example, headaches, stomach issues, or muscle pain (Schaufeli *et al*. 2020). The few studies examining health and psychosomatic complaints among school principals during the pandemic include the Asian and European partner countries of the Global Health Literacy Research Network (GLOBHL) that conducted the COVID-19 Health Literacy School Principal Survey (COVID-HL school survey). The Danish COVID-HL school study showed that higher workload of principals was associated with increased psychosomatic complaints (Kristensen *et al*. 2024), while the Pakistani study showed that the general health status was positively associated with fewer psychosomatic complaints (Zakar *et al*. 2024). Muscle pain was reported by almost half of the principals in the Polish study and headaches by one-fourth, with higher rates of psychosomatic complaints among older and female principals (Leksy *et al*. 2023b). In addition, the findings show that principals perceived helplessness—as one dimension of work-related stress—was a predictor for psychosomatic complaints and exhaustion due to increased workload, while increased self-efficacy was negatively associated with exhaustion.

Although burden and strain often take primacy, positive health indicators also merit attention in the context of health promotion (McQueen and Noack 1988). In public health, promoting well-being alongside disease prevention is a key objective (World Health Organization 2023). WHO defines well-being as a positive state, a resource in everyday life, which is influenced by social, economic, and environmental factors (World Health Organization 2021). While research on school principals in the context of health promotion remains limited, studies suggest that principals’ mental health and well-being vary according to work demands, work resources, sociodemographic characteristics, and school-level factors (Dadaczynski *et al*. 2022a, Doyle Fosco 2022, Tahir *et al*. 2026, Wang 2024). For example, older school principals reported higher well-being and fewer psychosomatic complaints than younger principals (Dadaczynski *et al*. 2022b), while another study found that female principals had lower well-being than male principals (Phillips *et al*. 2008). Country-level findings from the COVID-HL school survey indicated lower well-being among one-fourth of principals in Denmark (Kristensen *et al*. 2024), and approximately two-thirds in the UK (Wales and Northern Ireland) (Marchant *et al*. 2024), and Italy (Delbosq *et al*. 2026). The Taiwanese study reported that higher well-being of school principals was associated with lower vaccine hesitancy, suggesting well-being as an important target for emergency preparedness (Duong *et al*. 2021).

Several risk and protective factors for mental health and psychological well-being have been identified and are well established (Arango *et al*. 2021, Heinsch *et al*. 2022). While factors like sex or age are non-modifiable, modifiable factors include alcohol consumption, smoking, or physical activity (Dragioti *et al*. 2022, Islam *et al*. 2023, Licher *et al*. 2019). Modifiable factors differ in the degree to which they can be positively altered through public health interventions (Arango *et al*. 2018). In the context of health promotion and disease prevention, health literacy (HL) has been widely discussed as a modifiable protective factor and general health asset (Coughlin *et al*. 2020, Mantwill *et al*. 2015, Nutbeam 2008). HL can be defined as the ability to access, understand, appraise, and apply health information to enable informed health decisions (Sørensen *et al*. 2012). It is shaped by structural and organizational factors, and is also linked to health communication (Parker and Ratzan 2010). Higher levels of HL have been associated with actively engaging in one’s own health, making healthy lifestyle choices, and improved health outcomes (Berkman *et al*. 2011a, 2011b, Elkin 2024, Fan *et al*. 2021, Zheng *et al*. 2018). Conversely, low HL has been associated with mental health problems and poorer mental health outcomes (Guo *et al*. 2023, Haeri-Mehrizi *et al*. 2024, Hermans *et al*. 2021). Additionally, some studies reported associations between higher HL and higher well-being (Lindert *et al*. 2023, Tokuda *et al*. 2009). To the authors’ knowledge, the only study researching school principals’ HL prior to the pandemic was conducted in Germany and linked lower HL with lower well-being and increased psychosomatic complaints (Dadaczynski *et al*. 2022b).

During the pandemic, COVID-19 HL of principals was among the primary focus goals of the GLOBHL partner countries. Principals with higher COVID-19 HL in Hong Kong were less confused when confronted with health information compared with principals with lower COVID-19 HL (Lau *et al*. 2022). Confusion about health information was linked to more stress and fear symptoms among Taiwanese school principals, while higher COVID-19 HL scores were associated with lower scores of stress, fear, and depression (Duong *et al*. 2022). The Danish COVID-HL school study showed that among principals, perceived increased workload during the pandemic and frequent emotional exhaustion were associated with lower COVID-19 HL levels (Kristensen *et al*. 2024). Findings from the Pakistani study suggest that higher levels of COVID-19 HL may contribute to reducing emotional exhaustion in relation to work (Zakar *et al*. 2024). The country-specific studies from Hong Kong and Taiwan reported that higher levels of COVID-19 HL among school principals were associated with lower vaccine hesitancy (Duong *et al*. 2021, Lau *et al*. 2022). Together, these findings underscore the vital role of HL as an asset for principals’ health outcomes, but HL asserts value beyond individual health outcomes.

In the context of the COVID-19 pandemic and, most likely, beyond it, managing complex health information and navigating challenging communication environments proved particularly relevant for school principals and is a key characteristic of their leadership skills (Dadaczynski *et al*. 2021a). Alongside their responsibility for school health promotion activities, school principals are also responsible for enabling HL interventions at school level (Kirchhoff *et al*. 2025, Okan and Winkler 2026), while their personal HL has been linked to higher implementation of school health activities (Betschart *et al*. 2022, Dadaczynski *et al*. 2020b, Meyer *et al*. 2025). Addressing HL as a lever for health promotion in the education sector seems, therefore, most plausible.

Based on previous findings on the associations between HL and mental health outcomes, exploring the role of protective factors in times of crisis needs particular attention. HL has been termed a ‘social vaccine’ and a resource for managing information during the pandemic and other crisis (Okan *et al*. 2023, Paakkari and Okan 2020). COVID-19 HL is a context-specific version of HL referring to health information linked to the pandemic (Meyer *et al*. 2025). Aside from the country-level findings of the COVID-HL school study, evidence on school principals’ HL and its potential as an asset remains limited. This gap is particularly evident for international data, which would enable an analytical lens to understand HL independently of country-specific results, thereby supporting the development of recommendations that transcend national contexts and exhibit greater general applicability. Therefore, the present study aims to determine school principals’ COVID-19 HL levels across eleven countries in Asia and Europe, and to examine the association of COVID-19 HL, sociodemographic characteristics, and school-level factors with the personal mental health-related outcomes of well-being and psychosomatic complaints.

## Methods

### Study design

The COVID-HL school survey was conducted by the GLOBHL network from 2021 to 2022 in eleven countries (Denmark, Germany, Greece, Hong Kong, Italy, Poland, Romania, Switzerland, Taiwan, Türkiye, and United Kingdom) (Dadaczynski *et al*. 2022b, Global Health Literacy Research Network 2024). The survey aimed to assess the work situation and stress, health information, COVID-19 HL, and health outcomes among school principals, deputies, and members of the school leadership team. Most countries used convenience sampling with different recruiting strategies (e.g. social media, e-mail lists, co-operations with school principal associations). Participants provided written informed consent before gaining access to the online survey. The study was approved by each respective country-specific Ethics Board except for Denmark and Switzerland, where no ethical approval was required. Additional information about data collection in all countries (e.g. recruitment strategies and survey periods) can be found in Supplementary Table S1.

### Measurement tools

The COVID-HL school scale documentation lists all variables assessed in the online survey (Dadaczynski *et al*. 2021b). For the present international analysis, the focus is on the following variables: COVID-19 HL, well-being, and psychosomatic complaints. Self-reported COVID-19 HL was assessed through the HLS-COVID-Q22. In-depth insight into the development and validation of the tool can be accessed elsewhere (Meyer *et al*. 2024, Okan *et al*. 2020). High reliability indices were found, i.e. person separation = 3.41, person reliability = 0.92, item separation = 20.08, and item reliability = 1.0. The 22-item instrument measures a comprehensive form of HL, consisting of four subscales: ‘accessing’ (6 items), ‘understanding’ (6 items), ‘appraising’ (5 items), and ‘applying’ (5 items) health information in the context of the COVID-19 pandemic. Participants rated how easy or difficult they perceived each item on a 4-point Likert-type scale (ranging from ‘very difficult’ to ‘very easy’), with higher scores indicating higher COVID-19 HL.

Well-being was measured using the WHO-5 Well-Being Index (Bech 2004). Participants rated their mental well-being over the past 2 weeks across 5 items on a 6-point scale (ranging from ‘at no time’ to ‘all of the time’). WHO-5 is a well-established, valid, and reliable tool that is used globally as a screening tool for depression with a higher score indicating higher well-being (Topp *et al*. 2015). Psychosomatic complaints were assessed by the secondary symptoms subscale of psychosomatic complaints within the Manual Burnout Assessment Tool (BAT)—Version 2.0 (Schaufeli *et al*. 2020). Participants indicated how often they suffer from physical symptoms that are caused by psychological problems like headaches or stomach issues across 5 items on a 5-point scale (ranging from ‘never’ to ‘always’). A higher score indicates fewer psychosomatic complaints. A comprehensive psychometric analysis for the whole BAT has been conducted in a representative Flemish and Dutch sample. There has not been an in-depth analysis of the individual scales. However, the secondary symptoms (psychological distress and psychosomatic complaints) showed good internal consistency with Cronbach’s α = .94 for Flanders and α = .94 for the Netherlands, respectively (Schaufeli *et al*. 2020).

### Data analysis

#### Univariate analyses and data exclusion

IBM SPSS Statistics Version 31 was used for data analysis. Descriptive statistics (*M*, SD, %) as well as univariate analyses for sociodemographic differences in COVID-19 HL, well-being, and psychosomatic complaints were computed using *t*-tests for dichotomized variables (e.g. sex and age) and Welch’s ANOVAs for categorical variables (e.g. school type), followed by Bonferroni *post-hoc* tests. For the COVID-19 HL level computation, *n* = 1429 participants were excluded from data analysis, resulting in a final sample of *n* = 7215 school principals. The reasons for exclusion were: (i) not being the target population (not a school principal, deputy, or member of the school leadership team; *n* = 58 participants) and (ii) the HLS-COVID-Q22 has not been filled out (*n* = 1371 participants). For the regression analysis with the outcome variable well-being (WHO-5 Well-Being Index), *n* = 2014 participants were additionally excluded, resulting in a final sample of *n* = 5201 participants. Participants were excluded if they: (i) did not answer the WHO-5 Well-Being Index (*n* = 211 participants), (ii) had missing values for the explanatory variables (sex: *n* = 47 participants, age: *n* = 568 participants, school type: *n* = 198 participants), (iii) worked at a school type that was not included in the regression analysis (vocational school: *n* = 171 participants, schools for students with special educational needs: *n* = 281 participants), and (iv) indicated unlikely numbers for weekly working hours (i.e. < 10 or >70 hours, *n* = 286 participants) and (v) weekly teaching load (i.e. >40 hours, *n* = 252 participants). School principals from vocational schools and schools for students with special educational needs were excluded based on the small number of cases. Valid responses for weekly working hours and weekly teaching load were based on plausibility criteria derived from typical time distributions (Groß Ophoff *et al*. 2023, Tulowitzki *et al*. 2023). For the regression analysis with the outcome variable psychosomatic complaints, *n* = 2286 participants were excluded, resulting in a final sample of *n* = 4929 participants. Participants were excluded if they: (i) did not answer the psychosomatic complaints scale (*n* = 564 participants), (ii) had missing values for the explanatory variables (sex: *n* = 46 participants, age: *n* = 565 participants, school type: *n* = 197 participants), (iii) worked at a school type that was not included in the regression analysis (vocational school: *n* = 144 participants, schools for students with special educational needs: *n* = 273 participants), and (iv) indicated unlikely numbers for weekly working hours (*n* = 276 participants) and (v) weekly teaching load (*n* = 221 participants). Denmark and the UK did not collect data on age for anonymity reasons. Therefore, both countries were excluded from the regression analyses. Switzerland did not collect data on the psychosomatic complaints scale and hence, has been excluded in the regression analysis on psychosomatic complaints. This reduced the regression analyses to eight countries for psychosomatic complaints and nine countries for well-being, which should be considered when interpreting the results.

#### COVID-19 HL levels

Rasch–Thurstonian thresholds have been applied to calculate HL levels in the present study, allowing a more precise estimation of latent HL levels compared with traditional sum score approaches (Meyer *et al*. 2025). The HLS-COVID-Q22 is a Likert-type rating scale with categorical items. Therefore, Rasch analysis was computed to convert categorical responses into linear measures (Boone *et al*. 2014). WINSTEPS^®^ software has been used, applying the Partial Credit Model for polytomous data since the HLS-COVID-Q22 comprises four subscales (Linacre 2023, Masters 1982). The outcome of the Rasch analysis was an interval-scaled linear person measure for every school principal that reflects school principal’s COVID-19 HL (Boone *et al*. 2014). Principals with high person measures typically rated items as (very) easy, which translates to high COVID-19 HL. Although Greece (21/22 items) and Switzerland (16/22 items) did not collect data on every HLS-COVID-Q22 item, both countries were included in the calculation of HL levels as reliable person measures can be calculated without having responded to all items (Boone *et al*. 2014). Person measures were calculated based on the items that were answered without imputation.

Rasch–Thurstonian thresholds, i.e. the 50% cumulative probability threshold between adjoining rating scale categories, served as the basis for calculating COVID-19 HL levels (Boone *et al*. 2014, Linacre 2023). Thresholds were computed for all 22 items and between all 4 rating scale categories (‘very difficult’ and ‘difficult’, ‘difficult’ and ‘easy’, ‘easy’ and ‘very easy’). In the case of having a person measure that is at the cut-off between two categories, for example ‘easy’ and ‘very easy’, that school principal would have a 50% probability of choosing either ‘easy’ or ‘very easy’. In the next step, median values were calculated for the three cut-offs based on all Rasch–Thurstonian thresholds of the 22 items (Boone *et al*. 2014). This resulted in 4 COVID-19 HL levels (Level 1 = ‘insufficient’, Level 2 = ‘problematic’, Level 3 = ‘sufficient’, Level 4 = ‘excellent’).

#### Regression analysis

Pearson correlations were conducted to assess the associations between explanatory and outcome variables. Effect sizes were interpreted as specified by Cohen (low: *r* = .1, moderate: *r* = .3, high: *r* = .5) (Cohen 2013). Multiple linear regression analyses were calculated to investigate the association between COVID-19 HL and mental health-related factors. Blockwise multiple linear regression analyses were conducted separately for the outcomes well-being and psychosomatic complaints. Data pre-processing was performed for the outcome variables, as both were measured using Likert-type rating scales with categorical item response categories (Boone *et al*. 2014). Rasch analysis was administered to transform the responses into linear measures (Linacre 2023). For each outcome variable, a separate Rasch analysis was computed using Andrich’s Rating Scale Model for polytomous data since both variables do not comprise subscales (Andrich 1978). Then, the interval-scaled linear person measures were used as outcome variable. The regression models were controlled for sociodemographic characteristics of the school principals, i.e. sex (male vs. female), age (metric variable), and school-level factors, i.e. school type (2 dummy variables: primary school, secondary school; reference category: schools with primary and secondary classes), weekly working hours (metric variable), and weekly teaching load (metric variable) in Block 1. In Block 2, COVID-19 HL (metric variable) was added.

Subsequently, it was determined whether the model assumptions of a multiple linear regression analysis were met (Urban and Mayerl 2018). Multicollinearity was examined using Pearson correlation and computing the variance inflation factor (VIF) as well as tolerance of all explanatory variables. Correlation of >|0.80| between two explanatory variables and VIF >10 or tolerance <0.1 of one explanatory variable were used as indicators for multicollinearity (Field 2018). Homoscedasticity was examined visually via scatterplot of the residuals against the fitted values of the regression model and analytically using the Breusch–Pagan test (Urban and Mayerl 2018).

Multicollinearity was not present. Pearson correlations were not higher than |0.36|. Furthermore, the highest VIF value was 4.080 and the lowest tolerance value was .245. For both regression analyses, the scatterplots of the residuals against the fitted values of the regression model showed changes in variance of the residuals. Both Breusch–Pagan tests were significant. Heteroskedasticity needed to be assumed. Therefore, the robust standard error HC3 was used to account for heteroskedasticity (Hayes and Cai 2007).

Additionally, a robustness check of the results was performed as the sample sizes varied greatly between countries. To ensure that the findings are not driven by one large country sample, data weighting was conducted, so that the data from each country represented the same weight (Fishbein *et al*. 2021). A simple random downsampling method was used (OECD 2009). The blockwise multiple linear regression analyses with the weighted models could replicate the findings with the unweighted models. Accordingly, the following reports unweighted results.

## Results

### Study population

Across all countries, *N* = 8644 individuals participated in the study (see Table 1). Due to differences in data collection methods (e.g. recruitment via professional networks vs. official channels) and survey periods, countries’ sample sizes vary considerably, e.g. from *n* = 169 (Greece) to *n* = 2526 (Poland). Two-thirds of participants were female (66.1%). The mean age was *M* = 50.8 years (*SD* = 8.0; range = 20–70 years). The majority of participants were school principals (79.0%), 21.0% were deputies or members of the school management team. Most participants worked at a primary school (47.1%) or at a secondary school (33.4%).

**Table 1:** Characteristics of study participants (*N* = 8644).

Item	Category	Percentage (%)	Frequency
Country	Denmark	2.5	220
	Germany	25.2	2181
	Greece	2.0	169
	Hong Kong	7.3	634
	Italy	10.7	925
	Poland	29.2	2526
	Romania	3.5	306
	Switzerland	6.3	545
	Taiwan	4.8	413
	Türkiye	4.7	402
	United Kingdom	3.7	323
Sex	Male	36.2	2809
	Female	63.7	4953
	No specification	0.1	8
Age^a^	≤45 years	23.4	1687
	46–55 years	46.1	3320
	≥56 years	30.4	2191
Position	School principal	79.0	6106
	Deputy	21.0	1626
School type	Primary school	47.1	3557
	Secondary school	33.4	2524
	Vocational school	2.7	201
	Schools for students with special educational needs	4.1	311
	Schools with primary and secondary classes	12.7	956

Some responses were missing for some demographic variables.

^a^Denmark and the UK did not collect data on age to ensure anonymity of participants.

### Univariate and bivariate analyses

Table 2 displays mean values and standard deviations of all key variables, e.g. COVID-19 HL, well-being, and psychosomatic complaints, stratified by sociodemographic characteristics. Welch’s ANOVA indicated that school principals from secondary schools showed significantly higher COVID-19 HL than school principals from primary schools and school principals from schools with primary and secondary classes (*F*(2, 2474.08) = 14.71, *P* < .001, *η*^2^ = 0.01). Although the effect size was very small, principals aged 51 years and older had significantly lower COVID-19 HL, (*t*(6638) = 3.05, *P* = .02, *d* = 0.08), whereas no sex differences were found. Male principals reported significantly higher well-being, (*t*(6955) = 12.95, *P* < .001, *d* = 0.32), and less psychosomatic complaints (*t*(6603) = 17.84, *P* < .001, *d* = 0.46). Older principals reported significantly higher well-being (*t*(6429) = −4.08, *P* < .001, *d* = −0.10), as well as lower psychosomatic complaints (*t*(6079) = −2.87, *P* = .004, *d* = −0.07). School principals from secondary schools showed significantly lower psychosomatic complaints than school principals from primary schools and school principals from schools with primary and secondary classes (*F*(2, 2104.42) = 25.09, *P* < .001, *η*^2^ = 0.01). Although Welch’s ANOVA was statistically significant for school type and well-being, *post hoc* tests revealed no significant differences between groups.

**Table 2:** COVID-19 HL, well-being, and psychosomatic complaints stratified by sociodemographic characteristics.

	Mean (SD)		
Category	COVID-19 HL (*n* = 7215)	Well-being (*n* = 7004)	Psychosomatic complaints (*n* = 6651)
Sex		*	*
Male	2.47 (2.40)	.25 (2.70)	1.73 (1.58)
Female	2.39 (2.32)	−.57 (2.47)	1.04 (1.45)
Age	*	*	*
≤50 years	2.48 (2.43)	−.42 (2.60)	1.22 (1.55)
≥51 years	2.31 (2.26)	−.16 (2.54)	1.33 (1.48)
School type	*	*	*
Primary school	2.29 (2.33)	−.34 (2.58)	1.14 (1.47)
Secondary school	2.64 (2.39)	−.18 (2.65)	1.45 (1.58)
Schools with primary and secondary classes	2.41 (2.32)	−.35 (2.47)	1.20 (1.67)

Some responses were missing for some demographic variables, *P < .05.

### COVID-19 HL levels

COVID-19 HL person measures ranged from −7.03 to 7.82 logits and the mean person measure was 2.42 logits (SD = 2.35). Using Rasch–Thurstonian thresholds, Threshold 1 (e.g. between ‘very difficult’ and ‘difficult’) was at −2.74 logits. Threshold 2 was at −0.76 logits. Threshold 3 was at 3.145 logits. Therefore, 0.1% of school principals were classified as having insufficient, 3.3% problematic, 65.7% sufficient and 30.9% excellent COVID-19 HL. Table 3 shows the COVID-19 HL levels by country. However, we refrain from rating COVID-19 HL levels of countries due to convenience sampling and differences in sample sizes across countries. The stratification by country is for reporting purposes only.

**Table 3:** COVID-19 HL levels by country.

	COVID-19 HL level count (%)			
Country (*n*)	Insufficient	Problematic	Sufficient	Excellent
Denmark (220)	–	5 (2.3)	115 (52.3)	100 (45.5)
Germany (2082)	–	71 (3.4)	1433 (68.8)	578 (27.8)
Greece (169)	–	5 (3.0)	87 (51.5)	77 (45.6)
Hong Kong (624)	1 (0.2)	33 (5.3)	502 (80.4)	88 (14.1)
Italy (889)	1 (0.1)	41 (4.6)	639 (71.9)	208 (23.4)
Poland (1504)	5 (0.3)	45 (3.0)	944 (62.8)	510 (33.9)
Romania (256)	–	2 (0.8)	117 (45.7)	137 (53.5)
Switzerland (364)	1 (0.3)	4 (1.1)	234 (64.3)	125 (34.3)
Taiwan (413)	1 (0.2)	7 (1.7)	289 (70.0)	116 (28.1)
Türkiye (402)	1 (0.2)	17 (4.2)	199 (49.5)	185 (46.0)
UK (292)	–	10 (3.4)	179 (61.3)	103 (35.3)
Overall (7215)	10 (0.1)	240 (3.3)	4738 (65.7)	2227 (30.9)

Threshold 1 was at −2.74 logits, threshold 2 at −0.76 logits and threshold 3 at 3.145 logits.

### Pearson correlations

In Table 4, Pearson correlations for the multiple linear regression analyses are listed. The explanatory variables COVID-19 HL (*r* = .20) and age (*r* = .05) were significantly positively associated with the outcome variable well-being, while sex (*r* = −.17), school type (*r* = −.03), weekly teaching load (*r* = −.13), and weekly working hours (*r* = −.12) were nega­tively associated. Position showed no association with the outcome variable. There were no correlations above |*r*| = 0.34 among the explanatory variables. For the sec­ond multiple regression analysis, the explanatory variables COVID-19 HL (*r* = .17) and age (*r* = .06) were significantly positively associated with the outcome variable psychosomatic complaints, while sex (*r* = −.22), position (*r* = −.04) and weekly working hours (*r* = −.05) had significant nega­tive associations. There were no significant associations between school type, weekly teaching load and the outcome variable. The explanatory variables showed no to moderate correlations among each other (|*r*| = 0.00–0.35).

**Table 4:** Pearson correlations of explanatory and outcome variables.

	1	2	3	4	5	6	7	8
1 Well-being	–							
2 Sex	−.17*	–						
3 Age	.05*	.02	–					
4 Position	.01	.01	−.34*	–				
5 School type	−.03*	−.08*	.06*	−.05*	–			
6 Teach load	−.13*	.12*	−.24*	.32*	−.27*	–		
7 Work hours	−.12*	−.03*	.05*	−.11*	.13*	−.06*	–	
8 HL	.20*	−.03*	−.05*	.02	.00	−.05*	−.01	–
1 PC	–							
2 Sex	−.22*	–						
3 Age	.06*	.03*	–					
4 Position	−.04*	.00	−.35*	–				
5 School type	.01	−.06*	.05*	−.05*	–			
6 Teach load	.00	.11*	−.25*	.31*	−.27*	–		
7 Work hours	−.05*	−.02	.05*	.12*	.12*	−.06*	–	
8 HL	.17*	−.04*	−.06*	.03	.00	−.05*	−.01	–

HL, COVID-19 health literacy; PC, psychosomatic complaints.

^*^ *P* < .05, two-tailed tests.

### Regression analyses

The results of the blockwise multiple linear regression analysis with the outcome variable well-being are shown in Table 5. In Block 1, all explanatory variables have shown to be significant factors associated with well-being. When adding COVID-19 HL to the model in Block 2, this result remains, with sex (*B* = −.830, *P* < .001), age (*B* = .021, *P* < .001), position (*B* = .337, *P* < .001), primary school (*B* = .669, *P* < .001), secondary school (*B* = .481, *P* < .001), weekly teaching load (*B* = −.042, *P* < .001), weekly working hours (*B* = −.027, *P* < .001) and COVID-19 HL (*B* = .205, *P* < .001) acting as significant predictors. The overall regression model was statistically significant, explaining 9.6% of variance of well-being by the explanatory variables, with 3.4% explained by COVID-19 HL in Block 2, indicating a modest explanatory power of the model (adjusted *R*^2^ = 0.096, *F*(8, 5200) = 70.410, *P* < .001).

**Table 5:** Multiple linear regression analysis.

	*B*	SE	*P*	Tolerance	VIF	*R* ^2^	Adj. *R*^2^	Δ*R*^2^	*P*
Block 1						0.063	0.062		
Constant	.926	.392	.018						
Sex	−.836	.077	<.001	.943	1.061				
Age	.017	.005	<.001	.862	1.159				
Position	.348	.097	<.001	.794	1.259				
Primary school^a^	.669	.127	<.001	.263	3.801				
Secondary school^a^	.605	.129	<.001	.269	3.720				
Teach load	−.046	.005	<.001	.810	1.235				
Work hours	−.028	.004	<.001	963	1.038				
Block 2						0.098	0.096	0.034	<.001
Constant	.275	.381	.471						
Sex	−.830	.075	<.001	.943	1.061				
Age	.021	.005	<.001	.860	1.162				
Position	.337	.095	<.001	.794	1.259				
Primary school^a^	.619	.128	<.001	.263	3.804				
Secondary school^a^	.481	.130	<.001	.268	3.736				
Teach load	−.042	.005	<.001	.807	1.239				
Work hours	−.027	.004	<.001	.963	1.038				
COVID-19 HL	.205	.017	<.001	.986	1.014				

Outcome: well-being.

HL, health literacy.

^a^Primary school and secondary school were added as dummy variables with schools with primary and secondary classes as reference category.

Table 6 displays the results of the blockwise multiple linear regression analysis with the outcome variable psychosomatic complaints. In Block 1, all explanatory variables were significant predictors for psychosomatic complaints except for working at a primary school. This result remains in Block 2 when adding COVID-19 HL to the model, with sex (*B* = −.649, *P* < .001), age (*B* = .017, *P* < .001), position (*B* = −.187, *P* < .001), secondary school (*B* = .205, *P* = .018), weekly teaching load (*B* = .014, *P* < .001), weekly working hours (*B* = −.009, *P* < .001), and COVID-19 HL (*B* = .105, *P* < .001) being significant predictors. The overall regres­sion model was statistically significant, explaining 8.9% of variance of psychosomatic complaints by the explanatory variables, with 2.7% explained by COVID-19 HL in Block 2, indicating a modest explanatory power of the model (adjusted *R*^2^ = 0.089, *F*(8, 4928) = 61.480, *P* < .001).

**Table 6:** Multiple linear regression analysis.

	*B*	SE	*P*	Tolerance	VIF	*R* ^2^	Adj. *R*^2^	Δ*R*^2^	*P*
Block 1						0.064	0.062		
Constant	2.082	.237	<.001						
Sex	−.655	.047	<.001	.942	1.061				
Age	.015	.003	<.001	.853	1.172				
Position	−.180	.058	.002	.793	1.260				
Primary school^a^	.011	.086	.896	.245	4.077				
Secondary school^a^	.269	.088	.002	.249	4.020				
Teach load	.012	.003	<.001	.812	1.231				
Work hours	−.009	.002	<.001	.965	1.037				
Block 2						0.091	0.089	0.027	<.001
Constant	1.730	.233	<.001						
Sex	−.649	.046	<.001	.942	1.061				
Age	.017	.003	<.001	.851	1.176				
Position	−.187	.057	<.001	.793	1.260				
Primary school^a^	−.017	.085	.838	.245	4.080				
Secondary school^a^	.205	.087	.018	.248	4.036				
Teach load	.014	.003	<.001	.809	1.237				
Work hours	−.009	.002	<.001	.964	1.037				
COVID-19 HL	.105	.010	<.001	.985	1.015				

Outcome: psychosomatic complaints.

HL, health literacy.

^a^Primary school and secondary school were added as dummy variables with schools with primary and secondary classes as reference category.

## Discussion

The aims of the study were to gather first-time international data on school principals’ COVID-19 HL in Asian and European countries and to examine whether COVID-19 HL was associated with the personal mental health-related factors well-being and psychosomatic complaints. Overall, school principals had relatively high COVID-19 HL levels across all eleven countries. A positive associa­tion between COVID-19 HL and well-being as well as psychosomatic complaints was found through regression analyses, even after controlling for sociodemographic and school-level variables.

### International COVID-19 HL levels of school principals

In the current study, 0.1% of school principals had insufficient COVID-19 HL, 3.3% problematic, 65.7% sufficient, and 30.9% excellent. This finding indicates high HL of principals across countries as most respondents reported perceiving the items as either ‘easy’ or ‘very easy’. Examining country-specific results on principals’ personal HL, the findings from Denmark, Hong Kong, Pakistan, Poland and Türkiye (Kristensen *et al*. 2024, Lau *et al*. 2022, Leksy 2023; Öztürk *et al*. 2023, Zakar *et al*. 2024) showed that although the majority of principals in these countries reported high HL levels, a considerable proportion in each country also reported low levels. These discrepancies between country-specific and international analyses arise from methodological differences (Meyer *et al*. 2025). The international analysis calculated scores using Rasch–Thurstonian thresholds, whereas the country-specific results applied traditional cut-offs that were not derived from the data. While the novel method to compute HL levels has advantages over the commonly used technique (Boone *et al*. 2014, Meyer *et al*. 2025), comparing the HL levels to previous studies is not applicable because of the different techniques of setting cut-offs between levels. However, the methodological shift necessitates revisions to this HL questionnaire and its items, and probably to HL questionnaires in general, to establish a broader range between easy and difficult items. The HLS-COVID-Q22 appears to have predominantly easy items, a finding that warrants attention, particularly given that the data rely on participants’ self-reported perceptions of health information-related tasks. Therefore, a revision of currently used HL tools is necessary, preferably using Rasch analysis already in the developmental stage (Fleary *et al*. 2022, Fleary and Millington 2025, Meyer *et al*. 2024), which is in line with previous methodological discussions about the suitability of existing HL instruments and the need for further developing HL tools (Wirtz and Soellner 2022).

Nevertheless, findings from Germany confirm higher HL among school principals than in the general population (Dadaczynski *et al*. 2022b, Kolpatzik *et al*. 2025). This finding may be explained by school principals’ as an occupational group that is well-educated and high-earning (Dadaczynski *et al*. 2022b), factors considered social determinants of HL (Stormacq *et al*. 2019). An additional explanatory factor for the elevated COVID-19 HL levels observed in school principals may be volume, intensity, and clarity of pandemic-related communication (Okan *et al*. 2020). Official information and behavioural recommendations through public health authorities, institutions, and governments were often based on health-literate principles (e.g. plain language, pictures, videos) and, therefore, easier to access, comprehend, evaluate, and use (Hange *et al*. 2022, Saleem and Jan 2024). Although one-third of school principals in the present study demonstrated excellent COVID-19 HL, this figure is lower than anticipated given their educational and socioeconomic advantages over the general population. This finding shed light on a critical need: even an occupational group presumed to have excellent HL skills requires targeted support to foster them further. While universally targeted information campaigns would be advantageous for the general population, in the context of education and specifically for school principals, information that is distributed through communication channels available for them should be based on health-literate principles, e.g. through education ministry message boards, newsletters, or school principals’ associations.

School principals from secondary schools reported significantly higher COVID-19 HL than principals from primary schools and principals from schools with primary and secondary classes. In contrast to the current findings, another study from Germany conducted prior to the pandemic using a different school type classification found no significant differences in general HL based on school type (Dadaczynski *et al*. 2020b). However, while that study compared school types within Germany, the current study draws on international data from eleven countries in Europe and Asia, providing a more coherent picture of school principals’ HL status that can be used for intervention development. Comparing different school types across countries is difficult as education systems vary considerably globally (Cancarevic *et al*. 2021). In the absence of comprehensive research on differences by school type, comparisons seem currently most appropriate within a country or across countries with similar education systems.

Younger principals showed higher COVID-19 HL, while no sex differences were found. This result contrasts with a study among German school principals’ HL preceding the pandemic, which found no age differences but did report sex differences with males having lower HL (Dadaczynski *et al*. 2022b). International COVID-HL school findings are broadly consistent with country-specific findings (Kristensen *et al*. 2024, Lau *et al*. 2022, Leksy 2023, Zakar *et al*. 2024). The only exception to this pattern is Türkiye, where a significant sex difference emerged, with male principals demonstrating higher levels than female principals (Öztürk *et al*. 2023). Although most studies do not report sex differences in diverse populations (García-García and Pérez-Rivas 2022, Joveini *et al*. 2019, Veladas *et al*. 2023), other studies suggest higher HL in women (Kolpatzik *et al*. 2025, The HLS19 Consortium of the WHO Action Network M-POHL 2021, Yu *et al*. 2025). Women tend to be more aware about their health and show more frequent preventive behaviour (Hiller *et al*. 2017). Therefore, higher HL in women may reflect a greater intrinsic interest in learning about health topics. Greater knowledge could lead women to feel more confident in their ability to manage health information.

Current research on age differences in HL is mixed, with some studies finding lower levels among older individuals (Berens *et al*. 2016, Kobayashi *et al*. 2016) and others among younger individuals (Kolpatzik *et al*. 2025, Yu *et al*. 2025). This might partly be explained by differences in age classifications used for data analysis across these studies, as well as by different underlying HL definitions and measurement tools, making comparisons difficult. The M-POHL Survey of adults in 17 European countries found that the association between age and HL seems to be nonlinear, showing (inverted) U-curves in several countries (The HLS19 Consortium of the WHO Action Network M-POHL 2021). A possible explanation for higher COVID-19 HL among younger principals is that during the pandemic, COVID-19 information was particularly spread online (Krawczyk *et al*. 2021). Younger people not only tend to use online news more often (Boulianne and Shehata 2022), they also seem to have higher digital HL (Estrela *et al*. 2023), while digital HL is correlated with HL (Van Der Vaart and Drossaert 2017). Future studies should investigate under which conditions HL differences in age occur, preferably across sociodemographic characteristics. This would support the development of targeted interventions aimed at strengthening HL in diverse populations.

### Well-being and psychosomatic complaints

In the regression models, all explanatory variables were statistically significant predictors for well-being. Higher well-being was associated with male principals, older principals, being a vice principal or a member of the school management team, having a lower teaching load, less working hours, working at a primary or a secondary school compared with schools with primary and secondary classes, and higher COVID-19 HL. Fewer psychosomatic complaints were associated with male principals, older principals, being a school principal, having a higher teaching load, less working hours, working at a secondary school compared with schools with primary and secondary classes, and higher COVID-19 HL.

The current findings on sex- and age-related differences are partly consistent with previous research. While some studies have found lower well-being in female school principals (Dadaczynski *et al*. 2022b, Durrani and Makhmetova 2024, Phillips *et al*. 2008, Theodosiou *et al*. 2026), and one study found that they experience more psychosomatic stress than male principals (Dicke *et al*. 2018), other studies did not find sex-related differences for well-being (Dadaczynski and Paulus 2016) and psychosomatic complaints (Dadaczynski *et al*. 2022b). A meta-analysis revealed that female employees tend to report more work-related emotional exhaustion than male employees (Purvanova and Muros 2010). The multiple roles of women, e.g. domestic chores or childcare, can be contributing factors to work-related stress (Gyllensten and Palmer 2005). During the pandemic, females were more negatively affected by employment relations than males, followed by a shift to a more classical ‘gender-role’ (Reichelt *et al*. 2021). Therefore, female school principals’ mental health might have suffered more from additional stress imposed by the pandemic to balance their work-life responsibilities. Additionally, studies from Germany support the finding that older school principals reported higher well-being (Dadaczynski *et al*. 2022b, Dadaczynski and Paulus 2016) and fewer psychosomatic complaints (Dadaczynski *et al*. 2022b). The current findings might be explained by work experience. Older school principals could feel more confident in their abilities based on previous experiences on the job, which led to higher well-being (Dadaczynski and Paulus 2016). Higher well-being was associated with being a vice principal or a member of the school management team. In contrast, fewer psychosomatic complaints were reported among school principals. Dadaczynski *et al*. (2022b) found no differences in well-being and psychosomatic complaints in relation to the professional role. Research on school principals’ health has been neglected for a long time and is still limited (Dadaczynski *et al*. 2022a, Dadaczynski and Paulus 2015). Findings on sociodemographic characteristics and school-level factors associations with mental health-related outcomes are inconclusive and should be systematically investigated in future studies.

In the current study, a lower teaching load was associated with higher well-being whereas a higher teaching load was associated with fewer psychosomatic complaints. Phillips *et al*. (2008) suggested that a higher teaching load might facilitate positive feelings, as school principals might feel better equipped for teaching rather than applying leadership tasks. On the other hand, school principals have competing responsibilities and tasks (Gümüş *et al*. 2024). It could be argued that a higher teaching load can be considered as a burden alongside other duties and, therefore, negatively impact their mental health. Further research is needed to investigate how school principals’ teaching load and other leadership tasks affect their mental health. Less weekly working hours were associated with higher well-being and fewer psychosomatic complaints. Although not statistically significant, increased working hours have been linked to greater work-related stress (Phillips *et al*. 2008). Work-related stress is a negative predictor for well-being (Hirschle and Gondim 2020). Country-specific findings of the COVID-HL school survey showed that 74.4% of school principals in Germany (Dadaczynski *et al*. 2021a) and 67.2% of school principals in the UK (Marchant *et al*. 2024) reported that their weekly working hours were higher than prior to the pandemic. Thus, the current findings might be explained by the challenges that the COVID-19 pandemic imposed on school principals, leading to longer working hours, thereby increasing their perceived stress, which in turn might have affected their well-being and caused more psychosomatic complaints.

Fewer psychosomatic complaints were found for school principals from secondary schools, and higher well-being for respondents from primary and secondary schools compared with school principals from schools with primary and secondary classes. The German study by Dadaczynski *et al*. (2022b) found no school type differences for psychosomatic complaints but well-being varied according to the school type. The current findings may be partly explained by differences in sex distributions across school types. In the present international study, approximately two-thirds of the principals at a primary school and at schools with primary and secondary classes were female, whereas in secondary schools, the proportion was closer to half. Since female principals reported more frequent psychosomatic complaints, this would be consistent with the finding that principals from secondary schools reported less psychosomatic complaints. However, this would not explain the findings for differences in well-being by school type. Some studies suggest that primary school principals seem to have lower well-being (Dadaczynski and Paulus 2016, Phillips *et al*. 2008), while another study found that principals from grammar schools showed lower well-being (Dadaczynski *et al*. 2022b). It has been suggested that primary schools are usually smaller with less opportunities for task delegation, leading to lower well-being (Dadaczynski and Paulus 2016, Phillips *et al*. 2008). Since direct comparisons of school types across countries are difficult, future studies should investigate whether specific school attributes (e.g. school size, number of management team, and centralization) have an impact on school principals’ health. Although psychosomatic complaints are secondary symptoms of burnout, they could be the first warning signs of burnout (Hammarström *et al*. 2023, Schaufeli *et al*. 2020). There are several personal and organizational factors of school principal turnover for example school performance and accountability policy (Snodgrass Rangel 2018). While school principals are expected to achieve competing leadership responsibilities (Gümüş *et al*. 2024), they often lack the resources to do so (Kirchhoff *et al*. 2025). This leads to a work environment where high work demands are in contrast with the given conditions, which are antecedents for burnout (Rogers *et al*. 2025) and therefore, burnout is suggested as a contributing factor for turnover itself (DeMatthews *et al*. 2021). However, a stable leadership has been linked to student achievement and teacher turnover (Snodgrass Rangel 2018). This highlights the need to foster school principals’ mental health for their personal career and in their leadership role, for successful school development.

In the current study, higher COVID-19 HL has been found to be linked with higher well-being and fewer psychosomatic complaints. These findings are supported by a study conducted prior to the pandemic (Dadaczynski *et al*. 2022b). In addition, country-level findings of the COVID-HL school survey have shown that school principals’ COVID-19 HL was found to be associated with additional health-related outcomes, e.g. stress, depressive symptoms, emotional exhaustion, and vaccine hesitancy (Duong *et al*. 2021, 2022, Kristensen *et al*. 2024, Lau *et al*. 2022; Öztürk *et al*. 2023). Furthermore, COVID-19 HL as well as general HL revealed to be significant predictors for the implementation of health promotion in schools across different countries (Betschart *et al*. 2022, Dadaczynski *et al*. 2020b, Leksy *et al*. 2024, Meyer *et al*. 2025).

Combining these findings, HL seems to be an asset for school principals’ health and, at the same time, a determinant of school health promotion. As part of their leadership role, school principals are seen as gatekeepers and enablers for health promotion and prevention and for becoming a health-literate organization (Dadaczynski *et al*. 2022a, Kirchhoff *et al*. 2025). In this context, Dadaczynski *et al*. (2022a) proposed three different aspects: self-related health-promoting leadership, staff-related health-promoting leadership, and intervention-related health-promoting leadership. At the moment, there is no competence framework for health-promoting leadership and no systematic integration of health in school principal training and development (Leksy *et al*. 2024). HL should be discussed as a competence within a future health-promoting leadership framework, as it seems not only important for school principals in their own right but for the school at whole. This is especially important given a recent study on organizational HL in schools, which identified resource constraints as a key finding, including financial, time, and personnel shortages (Kirchhoff *et al*. 2025). These structural barriers undermine the conditions necessary for school principals to successfully implement health-promoting or health-literate frameworks at school. A systematic approach to strengthening school principals’ HL should embed health as a mandatory part of their training and development, supported by adequate resources that enable them to promote health within their schools.

### Limitations

Given the cross-sectional nature of this study, we can only report associations but cannot draw any causal inferences. Since most countries used convenience sampling, the present findings are not representative for specific countries or school principals in general. The variation in sample sizes led to disparate representation of countries in the analysis. However, using a weighted model in additional analyses replicated the current findings. Given the convenience sampling, differences in data collection and varying sample sizes across countries, the authors refrained from direct country comparisons. Instead, cross-country analyses are reported, focusing on general trends in school principals’ COVID-19 HL and mental health-related factors. To ensure participants’ anonymity, Denmark and the UK did not collect age data. Since age was an explanatory variable in the regression analyses, both countries were excluded from the data analysis. Switzerland did not collect data on psychosomatic complaints and was additionally excluded from this regression analysis. Thus, eight out of the eleven countries are represented in the main analysis. The HLS-COVID-Q22 was used to measure COVID-19 HL. Greece did not collect data on item 16, while Switzerland did not collect data on items 4, 6, 7, 11, 14, and 20. Even though reliable person measures can be computed using Rasch analysis when not all items of a questionnaire are answered by participants (Boone *et al*. 2014), the systematic missing of specific items from certain countries might have slightly affected the calculation of COVID-19 HL levels. The surveys were administered in the respective language of the countries. Some scales were already validated in these languages and if not, most countries used the translation-back-translation method. However, the translation processes could have been more systematic across countries. Additionally, government responses and emergency measures during the COVID-19 pandemic varied greatly between countries (Nurmandi *et al*. 2022). These differences in public health mitigation measures might have influenced COVID-19 HL and the other measures used in the present study. Social desirability, e.g. self-deception or social norm pressure, cannot be ruled out in self-report surveys (Larson 2019). School principals might have felt pressured to answer socially acceptable, for example reporting less psychosomatic complaints as mental health conditions are still widely stigmatized (Sickel *et al*. 2014). In the regression analyses, the additional variance explained by COVID-HL has been quite low (i.e. around 3%). Mental health is a complex topic with several influencing factors. Future studies should consider the interaction of multiple factors.

## Conclusion

Despite the cross-sectional study design, this is the first study that presents combined cross-national findings on school principals’ HL in Europe and Asia. Generally, principals demonstrated high levels of COVID-19 HL. Higher COVID-19 HL was associated with greater well-being and fewer psychosomatic complaints. Collectively, these findings underscore the importance of HL for both, school principals’ personal health and mental health and the health of their schools during times of crisis. A systematic approach to strengthening HL among school principals, and ideally embedded, e.g. in training and professional development, may be especially beneficial for female and younger principals, who reported lower well-being and more frequent psychosomatic complaints.

## Supplementary Material

daag098_Supplementary_Data

## Data Availability

The dataset generated and analysed during the current study is not publicly available as data analyses have not been finished. When data analyses are completed, the dataset will be made publicly available. Until then, the data are available from the corresponding author upon reasonable request.
